# A Decade-Long Case Series Report on the Surgical Management of Complicated Umbilical Hernia in Patients with Decompensated Liver Cirrhosis Utilizing Incisional Negative Pressure Therapy

**DOI:** 10.3390/medicina61071262

**Published:** 2025-07-12

**Authors:** Miha Petrič, Danaja Plevel, Uroš Tršan, Blaž Trotovšek

**Affiliations:** 1 Department of Abdominal Surgery, University Medical Centre Ljubljana, 1000 Ljubljana, Slovenia; danaja.plevel@kclj.si (D.P.); uros.trsan@kclj.si (U.T.); blaz.trotovsek@kclj.si (B.T.); 2 Medical Faculty, University of Ljubljana, 1000 Ljubljana, Slovenia

**Keywords:** complicated umbilical hernia, liver cirrhosis, incisional negative pressure therapy

## Abstract

*Background and Objectives*. Umbilical hernia is particularly common among patients with liver cirrhosis, affecting about 20% of this group, compared to 3–8.5% in healthy individuals. This increased prevalence is mainly due to weakened abdominal fascia, elevated intra-abdominal pressure, and malnutrition. The rapid progression of umbilical hernias often leads to complications such as skin necrosis, perforation, and strangulation. Historically, patients with liver cirrhosis and complicated umbilical hernia have faced high morbidity and mortality rates. However, recent advancements in perioperative management, especially in controlling ascites, have improved outcomes in elective treatments. Despite these advancements, managing patients with decompensated liver cirrhosis and complicated umbilical hernia in emergency settings remain a significant surgical challenge. *Materials and Methods*: We conducted a retrospective review of patients treated for complicated umbilical hernia at the University Medical Centre Ljubljana from 2015 to 2024, using prospectively collected data. This analysis involved implementing hernioplasty combined with incisional negative pressure wound therapy (iNPWT) as part of the surgical protocol. The primary endpoint of our study was the rate of local complications, while the secondary endpoints included the rate of systemic complications and 90-day mortality. *Results*: We treated 28 consecutive patients with complicated umbilical hernia and liver cirrhosis. Local wound complications were observed in three (10.7%) patients. Systemic complications developed in 10 patients (35.7%). The median duration of hospitalization was 8 days (range: 5–29), and no readmissions were recorded within the 30-day period. Two (7.1%) patients died within 90 days. *Conclusions*: Our experience indicates that iNPWT, when combined with surgical repair, can be safely utilized, yielding outcomes comparable to elective hernia repairs, even in emergency contexts. Further randomized controlled trials are necessary to validate these findings and optimize treatment protocols.

## 1. Introduction

Umbilical hernia is a common abdominal wall hernia in patients with liver cirrhosis. The prevalence of umbilical hernia in patients with liver cirrhosis is approximately 20% [1], compared to 3–8.5% in a healthy adult population [2]. The mechanism of development of umbilical hernia is thought to involve the weakening of the abdominal fascia due to raised intra-abdominal pressure, a higher level of protein loss, and skeletal muscle loss from malnutrition, combined with raised intra-abdominal pressure from ascites [3,4,5,6]. Ascites seems to play a major role in the rapid growth of umbilical hernias in patients with liver cirrhosis, as patients with decompensated liver cirrhosis have a higher prevalence (40%) of umbilical hernias [4,7]. Complications can be life-threatening. Skin necrosis, perforation, and incarceration are the most common complications, potentially leading to secondary peritonitis and sepsis [7,8]. Treating complicated umbilical hernia in a patient with decompensated liver cirrhosis remains challenging and controversial [9]. Historical reports show high morbidity (up to 70%) and high mortality (up to 80%) after supportive care [10]. Emergency surgery is associated with a high mortality rate (6–20%) [10]. In recent decades, improved perioperative management has resulted in lower, yet still unacceptably high, incidence rates of morbidity and mortality [11]. Higher mortality rates are associated with complications such as aspiration, pulmonary compromise, myocardial infarction, pneumonia, and metabolic derangements [4]. The effective control of ascites with diuretics, a transjugular intrahepatic portosystemic shunt (TIPS), or abdominal paracentesis is crucial for reducing the incidence of ascites leakage, wound infection, and sepsis [4,12]. However, most of these procedures are invasive and carry risks such as kidney failure, bleeding, thrombosis, or infection. Negative pressure wound therapy has shown a beneficial effect in reducing the incidence of surgical site infection and wound dehiscence in patients with various risk factors undergoing abdominal wall surgery [13]. In this retrospective review, we present 28 consecutive high-risk patients with liver cirrhosis and complicated umbilical hernia treated with emergency hernia repair combined with incisional negative pressure wound therapy (iNPWT).

## 2. Materials and Methods

We conducted a retrospective review of patients treated for complicated umbilical hernia at the University Medical Centre Ljubljana from 2015 to 2024, using prospectively collected data. Our study included all consecutive patients with liver cirrhosis and complicated umbilical hernia admitted to our emergency department. For each patient, we gathered data on age, sex, American Society of Anesthesiologists (ASA) score, Child–Pugh–Turcotte (CPT) classification score, model for end-stage liver disease (MELD) score, albumin–bilirubin (ALBI) score, number of operations, operation-related complications, duration of hospital stay, 30-day readmission rate, and 90-day mortality rate. The primary endpoint of our study was the incidence of local wound complications. The secondary endpoint was the incidence of systemic complications and 90-day mortality.

### 2.1. Surgical Procedure

**Figure 1:**
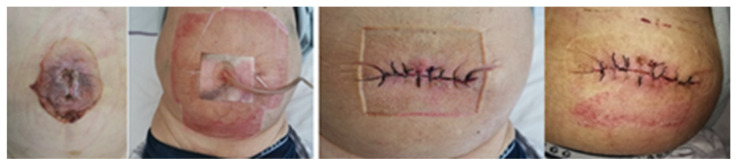
Approach in a patient with complicated umbilical hernia using iNPWT.

A short preoperative evaluation and restitution were performed in the hospital ward. Blood samples were collected and analyzed for potential electrolyte disturbances and signs of inflammation. Thromboelastography (TEG) analysis was employed for the targeted correction of blood coagulation if necessary. Patients received standard perioperative antibiotic prophylaxis with Cefazolin 2 g intravenously. Patients were placed in the supine position under general anesthesia and endotracheal intubation. A urinary catheter was then inserted. Routine nasogastric tube insertion was not performed, to prevent possible variceal bleeding. After thorough preparation, a skin incision was made around the umbilicus, and excision of the skin with the hernia sac was performed. Larger varices were ligated using 2-0 Vicrly™ (Ethicon, J&J Medtech, Cincinnati, OH, USA) sutures. The abdominal wall was sutured with interrupted 1 Vicrly™ (Ethicon, J&J Medtech, USA) sutures. Detailed hemostasis and suturing of the subcutaneous tissue were conducted with 2-0 Vicrly™ (Ethicon, J&J Medtech, USA) sutures. Wound infiltration with local anesthetics was performed, and the skin was closed with interrupted 2-0 Silk sutures (Figure 1).

### 2.2. Ascites Control

The routine use of abdominal drains was avoided and the preservation of as much volume of ascites as possible was attempted. A drain was used only if the ascites appeared to be cloudy or if signs of infection were present. Microbiological samples from ascites and necrotic wounds were routinely obtained.

### 2.3. Negative Pressure Wound Therapy

We placed V.A.C.^®^ Whitefoam™ Dressing (KCI, ACELITY V.A.C.^®^, San Antonio, TX, USA), size small, on surgical wounds with wide margins, and secured it with sterile tape (Figure 1). iNPWT was initiated immediately after skin closure, with a negative pressure of 125 mmHg applied through continuous suction. The foam was removed between days 5 and 7, and the underlying skin was inspected. Based on the local wound status and the fluid collected, another V.A.C.^®^ Whitefoam™ Dressing was used for an additional 5 to 7 days on the ward, at the discretion of the treating surgeon.

Initially, we excised white foam to fit directly on the suture line. However, based on personal communications with authors at various meetings, we employed broader (small size) foam, as suction reduces tissue edema, improves tissue perfusion, and removes excess fluid.

### 2.4. Postoperative Management

After surgery, the patients were admitted to the high-dependency unit for clinical stabilization and subsequently transferred to the hospital ward. We treated each patient using a multidisciplinary approach, which included early oral feeding and intensive physiotherapy. In cases of postoperative complications, we followed the standard clinical practices outlined in our department’s clinical pathway.

### 2.5. Statistical Analysis

The collected data were analyzed using SPSS for macOS, version 26. Descriptive variables such as frequency, percentages, mean/median, and range were used to describe and summarize the data.

## 3. Results

From 2015 to 2024, we surgically treated 28 consecutive patients with liver cirrhosis and complicated umbilical hernia. The patient characteristics are presented in Table 1.

In this retrospective review, most participants were men (27 individuals, 96.4%). The median age of the cohort was 62 years, with an age range of 40–89 years. A substantial proportion of patients (96.4%) were diagnosed with significant functional liver compromise according to the Child–Pugh classification, with 13 and 14 patients classified as Grade B and Grade C, respectively. All but one patient (C-P score A) had signs of ascites at the time of the operation, indicating signs of the decompensation of liver function. The median MELD score was 19.5 (range, 9–29), and 23 patients (82.1%) had MELD scores exceeding 15. The etiology of liver cirrhosis was predominantly attributed to ethanol, affecting twenty-six patients (92.8%), while two patients (7.1%) had cirrhosis due to HBV infection. Those that received a second application of iNPWT therapy while on the ward. The overall complication rate was 46.4% (13 of 28). A detailed list of patient complications is presented in Table 2.

In this retrospective review, local wound complications, such as infection or ascitic fluid leakage, were identified as the primary endpoints and were observed in three patients (10.7%). Systemic complications were observed in 10 patients (35.7%). The median duration of hospitalization was 8 days (range, 5–29 days), and no readmissions were recorded within the 30-day period. Furthermore, two patients (7.1%) died within 90 days.

Following discharge, patients were monitored by a dedicated hepatologist. In subsequent years, two (7.1%) patients experienced hernia recurrence, and two (7.1%) patients underwent liver transplantation.

## 4. Discussion

The surgical management of complicated umbilical hernias in patients with decompensated liver cirrhosis by utilizing iNPWT has been demonstrated to be both safe and effective. In a cohort of patients with liver cirrhosis classified with a Child–Pugh score of B or C, who are considered high-risk surgical candidates [10], we observed morbidity and mortality rates of 42.8% and 7.1%, respectively, in an emergency clinical setting for complicated umbilical hernia. Our findings are consistent with those reported in recent studies on elective hernia repair in patients with liver cirrhosis and uncomplicated umbilical hernia [8]. Notably, our mortality rate is significantly lower than the 19% reported in a large population-based study [14].

There are still unresolved issues in managing patients with liver cirrhosis and umbilical hernias. One major question is whether such patients should undergo surgical repair or be under surveillance. Data from a large population-based study revealed that only half of patients with liver cirrhosis and umbilical hernia were managed surgically, while the majority (86%) of healthy patients received surgical treatment [14]. A comparative study showed that conservative management was successful in less than a quarter of patients with liver cirrhosis and umbilical hernia, with a mortality rate of 15.3% (2 out of 13 patients) [12]. In surgically treated patients, morbidity reached 29.4% due to local wound complications (three patients) and hernia recurrence (four patients), with no mortality in this group [12]. This study has the potential for bias in patient treatment selection due to its retrospective nature.

Numerous reports indicate high morbidity and mortality rates in patients with liver cirrhosis undergoing emergency surgery for complicated umbilical hernia [14,15,16]. However, due to a better understanding and perioperative management of cirrhotic patients, the successful elective treatment of umbilical hernias has been reported, with acceptably low morbidity and mortality (0.6–2% vs. 3.8–20%) [4,10,17,18]. Another comparative study indicated that negative predictive factors for morbidity and mortality in cirrhotic patients include emergency repair, diabetes, chronic obstructive pulmonary disease, congestive heart failure, and liver cirrhosis [19]. Interestingly, patients with liver cirrhosis and umbilical hernia treated electively showed similar outcomes to healthy individuals [19]. Similar findings were reported in Korea, where elective treatment in cirrhotic patients resulted in overall morbidity and mortality rates of 42% and 6.5%, respectively [8]. One study identified hepatic reserve and the presence of ascites as negative prognostic factors in determining treatment type (surgery vs. conservative) [8]. In our study, systemic complications were present in 10 (35.7%) patients, while 3 (10.7%) developed local wound complications (ascitic leak or wound infection). In-hospital mortality occurred in two patients due to septic shock and liver failure.

For patients who are liver transplantation candidates, umbilical hernias should be corrected during the procedure [20]. A thorough assessment should be made for non-transplant patients. The risk of postoperative complications was assessed using different scoring systems. The ASA score is relatively unspecific for determining postoperative complication risk in end-stage liver disease patients, since they automatically receive a score of 3 or more, potentially undermining the risk of other factors [21]. The Child–Turcotte–Pugh (CTP) score correlates well with postoperative complication incidence, but is subjective in scoring hepatic encephalopathy and ascites [22]. Mortality in Child class B and C patients was 23% and 84%, respectively [22]. The MELD score, initially used as a prognostic tool for survival in patients undergoing TIPS, demonstrates good postoperative risk assessment for end-stage liver disease [23]. Each additional point between 5 and 20 increases mortality risk by 1% [24]. Liver coagulation disorders are best evaluated using thromboelastography (TEG), which provides real-time, dynamic insights into complex coagulation abnormalities in cirrhotic patients, outperforming traditional tests [25]. Cirrhotic patients with umbilical hernia, particularly those over 65 years, with MELD scores greater than 15, low albumin levels, high white blood cell counts, and low platelet counts, are poor surgical candidates [26,27]. Our study concentrated on a specific surgical cohort characterized by a high risk of post-procedural complications, with most patients presenting with decompensated liver cirrhosis (presence of ascitic fluid, 96.4%) and elevated MELD scores (>15 in 82.1% of patients). All patients underwent surgical intervention in the acute context of umbilical hernia complications, such as perforation and skin necrosis.

Ascites management is essential, influencing rapid umbilical hernia growth and the postoperative period, which affects wound complications and systemic infection. Medical treatment and paracentesis are recommended for patients awaiting umbilical hernia repair [7]. Cases of pleural effusion might require TIPS or intraoperative drainage [8]. However, peritoneal drain insertion is invasive, potentially causing abdominal wall bleeding, acute kidney failure, and ascending infection, significantly increasing morbidity and mortality [28]. TIPS complications like encephalopathy, shunt thrombosis, and liver dysfunction or failure often result in death [29]. Elective mesh hernia repair is safe, decreasing hernia recurrence [30,31]. One trial showed a significant recurrence drop (14.2% vs. 2.7%) when using mesh in patients with cirrhosis undergoing umbilical hernia repair, compared to those without mesh [32]. Despite its efficacy, using mesh in emergency repairs of complicated hernias is inadvisable [17,32]. Long-term follow-up of the patients indicated a relatively low rate of hernia recurrence, with only two patients (7.1%) experiencing recurrence necessitating elective mesh hernia repair. However, data for three patients were unavailable due to dropouts, although they remained alive at the time of writing this. Additionally, two patients successfully underwent liver transplantation. Also, no 30-day rehospitalizations were necessary due to systemic or local complications.

This is the first paper to describe iNPWT in cirrhotic patients with complicated umbilical hernias treated in emergency settings. Extensive evidence of iNPWT’s use after the surgical management of complicated umbilical hernia in liver cirrhosis is lacking. Negative pressure wound therapy (NPWT) is predominantly employed for wound closure via secondary intention or delayed closure, particularly in cases of surgical wound infection (SWI), dehiscence, or necrosis of the wound edges [33]. Another indication for its use is the infection of a mesh prosthesis [34]. NPWT facilitates the proliferation of granulation tissue through the mesh, potentially safeguarding it from bacterial colonization within the surgical wound [34]. Nevertheless, NPWT is effective in enhancing wound healing in cirrhosis and reducing surgical site occurrences, infections, wound dehiscence, and hernia recurrence in ventral hernia repair [35]. Early reports indicate iNPWT’s potential in reducing infection rates, seroma formation, and reoperation in the general population [36]. A review highlighted iNPWT’s effectiveness in lowering SSI rates without affecting wound dehiscence, although some experienced blistering and allergic reactions [37]. iNPWT has demonstrated efficacy as a cost-effective treatment option for abdominal wall wound dehiscence when compared to the standard approach [38]. This conclusion is supported by a cost–utility analysis that indicated the favorable cost-effectiveness of iNPWT [39]. In our study, three (10.7%) patients developed local wound complications, with one (3.5%) experiencing ascites leakage. In the no-drain group, only two (9.5%; 2/21) had superficial wound infections without ascites leakage, underscoring iNPWT’s positive effects on wound healing and preventing fistula development or infection.

Our study had several limitations. First, it employed a retrospective review of the case series results’ design. Secondly, the absence of patient randomization and the relatively small sample size may obscure significant factors. Additionally, due to the closure of our former city hospital and reliance on paper documentation, we were unable to compare our findings with the historical management of patients with liver cirrhosis and complicated umbilical hernias. Furthermore, given the positive outcomes observed, it was ethically untenable to manage this patient cohort using hernioplasty and routine ascitic drainage. In the authors’ opinion, preserving as much ascitic fluid as possible is crucial for patient benefit. We also demonstrated that our method of combining hernioplasty and iNPWT can be safely and efficiently employed in various clinical scenarios. It is crucial to recognize that the included patients underwent emergency procedures without full preoperative preparation or medical stabilization. The simplicity of this technique offers the prompt management of patients with liver cirrhosis and acute complications of umbilical hernias outside specialized tertiary centers. As we have demonstrated, this can significantly reduce patient mortality. Our patient group varied in terms of operation indications and pre-surgery physical states. To comprehensively assess the efficacy of the described technique, a randomized controlled trial is essential to fully evaluate the benefits of iNPWT.

## 5. Conclusions

The surgical management of complicated umbilical hernias in patients with decompensated liver cirrhosis can be safely and effectively achieved with iNPWT. The use of NPWT has significantly reduced the incidence of wound complications, such as inflammation and ascites leakage. By employing iNPWT, we were able to successfully prevent local complications arising from ascites. iNPWT is also significantly less invasive than procedures like TIPS and abdominal paracentesis. Given the high-risk nature of this surgical population, patients should be treated in a tertiary liver transplant center. Further studies are needed to thoroughly evaluate the use of iNPWT in patients with liver cirrhosis and umbilical hernias.

## Figures and Tables

**Table 1:** Patient characteristics; C-P (Child–Pugh) score.

male		27 (96.4%)
female		1 (3.6%)
Age (years)	median	62 (range: 40–89)
C–P score		
B		13 (46.4%)
C		14 (50%)
MELD score	median	19.5 (range: 9–29)
ALBI score	median	2 (range: 3)

**Table 2:** Detailed list of patient complications.

Complication	Number	%
Sepsis	2	7.1
Multiorgan failure	1	3.6
Liver failure/decompesation	3	10.7
Acute kidney failure	2	7.1
Wound infection	2	7.1
Ascitic leak	1	3.6
Delirium/somnolence	3	10.7
Symptomatic pleural effusion	1	3.6

## Data Availability

The data is stored in our institution’s database and is available upon request.

## Abbreviations

The following abbreviations are used in this manuscript:

iNPWT	Incisional negative pressure wound therapy
TIPS	Transjugular intrahepatic portosystemic shunt
ASA	American Society of Anesthesiologists
CPT	Child–Pugh–Turcotte
MELD	Model for end-stage liver disease
ALBI	Albumin–bilirubin
